# Comparison of impulsivity in non-problem, at-risk and problem gamblers

**DOI:** 10.1038/srep39233

**Published:** 2016-12-15

**Authors:** Wan-Sen Yan, Ran-Ran Zhang, Yan Lan, Yong-Hui Li, Nan Sui

**Affiliations:** 1 Department of Psychology, School of Medical Humanitarians, Guizhou Medical University, Guiyang 550004, China; 2 Key Laboratory of Mental Health, Institute of Psychology, Chinese Academy of Sciences, Beijing 100101, China

## Abstract

As a non-substance addiction, gambling disorder represents the model for studying the neurobiology of addiction without toxic consequences of chronic drug use. From a neuropsychological perspective, impulsivity is deemed as a potential construct responsible in the onset and development of drug addiction. The objective of this study was to investigate the associations between impulsivity and gambling status in young adults with varying severity of gambling. A sample of 1120 college students, equally divided into non-problem, at-risk and problem gamblers, were administered multiple measures of impulsivity including the UPPSP Impulsive Behaviors Scale (UPPSP), the Barratt Impulsiveness Scale-11 (BIS-11), and the Delay-discounting Test (DDT). Compared with non-problem gamblers, both at-risk gamblers and problem gamblers displayed elevated scores on Negative Urgency, Positive Urgency, Motor Impulsiveness, and Attentional Impulsiveness. Problem gamblers showed higher scores than at-risk gamblers on Positive Urgency. Logistic regression models revealed that only Negative Urgency positively predicted both at-risk gambling and problem gambling compared to non-problem gambling. These results suggest that dimensions of impulsivity may be differentially linked to gambling behavior in young adults, with Negative Urgency putatively identified as an important impulsivity-related marker for the development of gambling disorder, which may provide a better understanding of the pathogenesis.

Impulsivity, a multidimensional trait that is viewed as a core pathological construct of many mental disorders^1^, is briefly defined as “the tendency to act prematurely without foresight”^2^. At the personality level, impulsivity is assessed using self-report questionnaires such as the Barratt Impulsiveness Scale (BIS)^3^, which involves three dimensions named Motor Impulsiveness, Attentional Impulsiveness, and Non-planning Impulsiveness. At the neuropsychological level, impulsivity is thought to arise from impaired inhibitory control taxed by cognitive tasks such as the Stop-Signal Test^4^. Mounting studies have suggested that higher impulsivity traits are closely linked to different forms of drug use and abuse, including stimulants, opiates and alcohol^5^,^6^,^7^,^8^,^9^. Impulsivity is also being considered a vulnerability marker for substance use disorders^9^,^10^,^11^,^12^, playing a crucial role in predicting onset and maintenance of drug taking and seeking as well as relapse rates^13^. However, given the diversity of the term impulsivity as well as its underlying neurobiological underpinnings, it has been suggested that impulsivity is not a unitary construct at all, but with common themes including decreased inhibitory control (impulsive disinhibition), intolerance of delay to rewards (delay discounting), quick decision-making due to lack of consideration (impulsive decision-making), and poor attentional ability (impulsive inattention)^14^,^15^. Considering the multifaceted nature of impulsivity, as well as the confusion effects of neurological sequelae in chronic drug use, it remains controversial whether impulsivity can be recognized as one of the effective potential endophenotypes for drug addiction.

Gambling disorder is currently the only non-substance condition listed as an addiction in the DSM-5^16^, sharing many similarities with drug addiction on neurobiological underpinnings^17^,^18^. As a non-substance addiction, gambling disorder does not necessarily involve the neurotoxicity associated with concomitant drug use, thus representing the model for studying the neurobiology of addiction without the toxic consequences of chronic drug use^19^,^20^. Although most people who engage in gambling do so responsibly, with the prevalence of gambling disorder ranging from 0.2% to 5.3% in general population^21^, some individuals become preoccupied with gambling and may finally evolve into problem gamblers^22^. As such, the relationship between gambling disorder and aspects of psychological traits (especially impulsivity traits) merits much attention^9^,^23^,^24^.

Previous case-control studies have compared dimensions of impulsivity between problem/pathological gambling (PG) individuals and healthy controls (see refs 9 and 25 for reviews). Indeed, results have demonstrated elevated self-report impulsivity scores in PG group such as on the BIS (e.g., Motor Impulsiveness, Attentional Impulsiveness, Non-planning Impulsiveness)^26^,^27^,^28^,^29^,^30^ and the UPPSP Impulsive Behaviors Scale (e.g., Negative Urgency, Positive Urgency)^31^,^32^,^33^,^34^,^35^,^36^. Neurocognitive investigations have also found that PG is associated with impaired inhibition performance on the Go/No Go Test^26^,^37^,^38^,^39^ and the Stop-Signal Test^27^,^32^,^40^,^41^,^42^,^43^. In addition to these cross-sectional data, there is some preliminary evidence that self-report impulsivity traits in adolescence are prospectively associated with gambling disorder in follow-up assessments^44^,^45^,^46^,^47^. However, negative results concerning the closed associations between impulsivity and gambling disorder have also been found (see refs 19, 28, 48 and 49). In consideration of the complexity of impulsivity itself and inconclusive causality between impulsivity and gambling disorder, alternative studies have been suggested to be designed to gather more empirical evidence^50^.

To better reflect the taxonomy of gambling behavior and elucidate the potential role of psychological and neurocognitive components in the development from recreational gambling to problem gambling, two recent studies used a non-dichotomous classification of gambling disorder comparing people with PG to those at an increased risk of developing PG (i.e., at-risk PG) as well as those with no risk of PG ((i.e., no-risk PG) on tasks of attentional bias^51^, and response inhibition and cognitive flexibility^52^. Impaired response inhibition and cognitive flexibility were found in PG compared with at-risk and no-risk PG, and differences on attentional biases were not significant between PG and at-risk PG, but significant between PG and no-risk PG. Despite these limited results, understanding the chain of progression from recreational gambling to problem gambling is vital for understanding the pathogenesis of gambling disorder^52^.

In the present study, we thus employed a non-dichotomous category of gambling disorder (i.e., no PG, at-risk PG, and PG) with a relatively large sample, aiming to further explore the relationships between different dimensions of impulsivity traits and gambling behavior using the Barratt Impulsiveness Scale (BIS), the UPPSP Impulsive Behaviors Scale, and a monetary-choice delay discounting test (DDT)^53^. It was generally hypothesized that different dimensions of impulsivity would be primarily correlated with different forms of gambling behavior. And when all variables were simultaneously entered into model, specific traits such as higher negative urgency^54^ would be evident prior to the development of overt pathology (i.e., elevated scores detected both in at-risk PG and PG compared with no PG, but no difference between at-risk PG and PG), representing a vulnerability marker or candidate of gambling disorder^52^, however, other dimensions of impulsivity might not be.

## Methods

### Participants and Procedure

Data for this study were collected in November 2014, which was part of a three-year longitudinal study investigating the relationships between impulsivity and gambling among college students enrolled at a large public university. Participants were 1180 young adults, who were recruited from 12 randomly-selected freshman classes. All freshman students were invited to provide demographic information and complete a battery of self-reported questionnaires in a 45-minute psychology class. Inclusion criteria included: (1) between 18 and 25 years of age, (2) willingness to participate in the longitudinal study, and (3) in-state residence. The exclusion criteria included the current/past major psychiatric disorders (schizophrenia, psychotic episodes, major depressive disorder or bipolar disorder), a history of brain injury/trauma, current/past neurological diseases or mental disorders, and current/past use of psychoactive substance (e.g. cocaine, heroin, methamphetamine, marijuana), assessed with the Structured Clinical Interview for DSM-IV disorders (SCID)^55^ by well-trained psychiatrists and clinical psychologists during the first three months (Aug. 31 to Nov. 30, 2014) of university enrollment. A total of 24 students who met one or more of these exclusion criteria were excluded from the study enrollment. Furthermore, all students were assessed with the Fagerström Test for Nicotine Dependence (FTND)^56^ and the Alcohol Use Disorders Identification Test (AUDIT)^57^, and those who scored ≥6 on the FTND (probable nicotine dependence) and/or scored ≥8 on the AUDIT (probable alcohol use disorders) were excluded (n = 36). Thus finally, 1120 participants (mean age = 19.13, ranging from 18 to 23 years) were included in this study. Only data from the first year of the longitudinal study (Wave 1) were analyzed in the present study. All subjects provided written informed consent and were compensated for their time with a gift equal to RMB ¥ 50. This study was reviewed and approved by the Human Research Ethics Committee at the Guizhou Medical University. Our proposed recruitment process, study design, and plans to compensate participants were consistent with the Declaration of Helsinki.

### Gambling Group Classification

Gambling group status was determined by using the South Oaks Gambling Screen (SOGS)^58^. In line with previous studies (e.g. refs 51 and 52), these were non-problem gamblers that scored 0–2 (n = 978), gamblers at risk with a score equal to 3-4 (n = 87), and problem gamblers that scored 5–20 (n = 55) with the maximum being 13 in the present sample. Most of the forms of gambling among the gamblers are mahjong, poker cards, and lottery, because casino games are legally prohibited in whole Mainland China, in accordance with that in our previous study^59^.

### Impulsivity Measures

The UPPSP Impulsive Behaviors Scale^60^,^61^ is a 59-item inventory designed to measure five distinct personality pathways to impulsive behavior: Sensation Seeking, Lack of Perseverance, Lack of Premeditation, Negative Urgency, and Positive Urgency. Items were rated on a 4-point scale from 1 (strongly agree) to 4 (strongly disagree). Total scores on each of the five UPPSP dimensions were obtained for analyses. In this study, we adopted the Chinese version of UPPSP^62^. This scale had a good fit of five-factor model (χ^2^/*df* = 2.26, GFI = 0.88, NNFI = 0.93, CFI = 0.93, RMSEA = 0.044) among college students. Cronbach’s α was 0.75–0.84 across all five subscales.

The Barratt Impulsiveness Scale-11 (BIS-11)^3^ is a 30-item scale consists of three dimensions: Motor Impulsiveness, Attentional Impulsiveness, and Non-planning Impulsiveness. Items were rated on a 4-point scale. Sum scores on each of the three subscales were obtained for analyses. Higher scores indicate higher impulsivity. In the present study, we used the Chinese version of BIS-11^63^. This scale showed good psychometric properties in college students. Cronbach’s α was 0.77–0.89 and test-retest reliability was 0.68–0.89 for the three subscales.

The Delay-discounting Test (DDT)^53^ is a monetary-choice questionnaire between smaller, immediate rewards and larger, delayed rewards. Delay discounting is a tendency that individuals prefer a smaller immediate reward to a larger delayed reward, which is termed impulsivity as opposed to self-control^64^. The DDT is composed of a set of 27 choices^53^. The degree of discounting was calculated by the following hyperbolic equation: *V* = *A*/(1 + *kD*). In this equation, *V* is the subjective value of the delayed reward, *A* is the nominal amount of the delayed reward, *D* is the length of the delay, and *k* is a free parameter that describes the degree of discounting. The larger the *k* parameter, the quicker the discounted value decreases over time. In our study, we employed a revised version used in Chinese cultural context^65^,^66^. This version has both gain- and loss- conditions. Examples for the gain condition are “*A: receiving ¥9500 now; B: receiving ¥10000 one year later*” and “*A: receiving ¥500 now; B: receiving ¥10000 one year later*”. Examples for the loss condition are “*A: losing ¥9500 now; B: losing ¥10000 one year later*” and “*A: losing ¥500 now; B: losing ¥10000 one year later*”. The discounting rate (*k*) was calculated for each condition. Consistent with previous literature, *k* scores were log-transformed to approximate a normal distribution.

### Data analysis

The data were analyzed with the Statistical Package for the Social Sciences for Windows, Version 15.0 (SPSS Inc., Chicago, IL, USA). Group differences in categorical data (i.e. gender, ethnicity, home locality) were analyzed with chi-square tests. Impulsivity scores were compared between the groups using a multivariate analysis of variance (mANOVA) model with age, gender, ethnicity, and home locality as covariates. Post-hoc comparisons were investigated using Fisher’s least significant differences protected t-test. Partial correlations were tested between impulsivity measures and SOGS scores, controlling for age, gender, ethnicity, and home locality. Logistic regression analyses were used to test the effects of impulsivity scores on gambling behavior, controlling for age, gender, ethnicity, and home locality. According to the variance inflation factor (VIF), multicollinearity was not a problem for any variable (VIF < 10) in the regression models. Significance was defined as *p* < 0.05, two-tailed.

## Results

### Group differences on Demographics and Impulsivity measures

A description of demographics and impulsivity scores across three gambling groups is presented in Table 1.

Consistent with the literature^16^, males were more likely than females to be problem gamblers (χ^**2**^ = 42.349, *p* < 0.001). Follow-up tests revealed that the rate of males was significantly lower in non-problem gamblers (NPGs) than in at-risk gamblers (ARGs) (χ^**2**^ = 11.682, *p* = 0.001) and in problem gamblers (PGs) (χ^**2**^ = 34.337, *p* < 0.001), and the rate of males in ARGs was lower than that in PGs (χ^**2**^ = 5.015, *p* = 0.025). No between-group differences were observed for ethnicity (χ^**2**^ = 3.920, *p* = 0.141) or home locality (χ^**2**^ = 2.436, *p* = 0.296). As expected, the NPGs displayed lower scores than ARGs and PGs both on the FTND (*F*_(2,1117)_ = 16.318, *p *< 0.001) and AUDIT (*F*_(2,1117)_ = 23.816, *p *< 0.001).

On the UPPSP, the mANOVA model revealed no significant group differences on Sensation Seeking (*F*_(2,1113)_ = 0.477, *p* = 0.620), Lack of Perseverance (*F*_(2,1113)_ = 2.126, *p* = 0.120) or Lack of Premeditation (*F*_(2,1113)_ = 1.631, *p* = 0.196), but on Negative Urgency (*F*_(2,1113)_ = 20.180, *p* < 0.001, η_p_^2^ = 0.035) and Positive Urgency (*F*_(2,1113)_ = 12.688, *p* < 0.001, η_p_^2^ = 0.022). Post-hoc tests revealed that PGs had higher scores than NPGs on Negative Urgency (M_d_ = 4.172, *p* < 0.001) and Positive Urgency (M_d_ = 4.233, *p* < 0.001), and ARGs also displayed elevated scores on both dimensions compared with NPGs (M_d_ = 2.466, *p* < 0.001; M_d_ = 1.879, *p* = 0.011, respectively). There was no significant difference between PGs and ARGs on Negative Urgency (*p* = 0.077), but PGs showed higher scores than ARG on Positive Urgency (M_d_ = 2.354, *p* = 0.039).

On the BIS-11, the group differences were significant on Motor Impulsiveness (*F*_(2,1113)_ = 10.519, *p* < 0.001, η_p_^2^ = 0.019), Attentional Impulsiveness (*F*_(2,1113)_ = 7.209, *p* = 0.001, η_p_^2^ = 0.013), and Non-planning Impulsiveness (*F*_(2,1113)_ = 3.852, *p* = 0.022, η_p_^2^ = 0.007). Post-hoc tests showed that both PGs and ARGs scored higher than NPGs on Motor Impulsiveness (M_d_ = 1.888, *p* < 0.001; M_d_ = 0.900, *p* = 0.015, respectively) as well as on Attentional Impulsiveness (M_d_ = 1.576, *p* = 0.001; M_d_ = 0.762, *p* = 0.041, respectively), and only PGs scored higher than NPGs on Non-planning Impulsiveness (M_d_ = 1.499, *p* = 0.018). No significant differences were found between PGs and ARGs on Motor, Attentional or Non-planning Impulsiveness scores (*p* = 0.082, *p* = 0.156, *p* = 0.378, respectively).

On the DDT, there were no significant group differences of *k* values (log-transformed) between NPGs, ARGs and PGs either in gain condition (*F*_(2,1113)_ = 0.538, *p* = 0.584) or in loss condition (*F*_(2,1113)_ = 2.409, *p* = 0.090).

### Partial Correlations between impulsivity measures and SOGS scores

Partial correlations between impulsivity measures and SOGS scores, controlling for age, gender, ethnicity, and home locality, were displayed in Table 2. Significant positive associations were detected between SOGS scores and UPPSP Negative Urgency, Positive Urgency, and BIS-11 Motor Impulsiveness, Attentional Impulsiveness, and Non-planning Impulsiveness scores (*r*_*p*_ = 0.112–0.213, *ps* < 0.001). However, no significant associations were found between SOGS scores and UPPSP Sensation Seeking, Lack of Perseverance, Lack of Premeditation, DDT *k* values (log-transformed) in gain condition (*k*_gain_) or in loss condition (*k*_loss_).

### Logistic regression outcomes

Logistic regression models were used to examine the effects of impulsivity dimensions on gambling behavior. Binary regression models were conducted comparing the groups each other (i.e., non-problem gambling VS at-risk gambling, non-problem gambling VS problem gambling, and at-risk gambling VS problem gambling). A 2-step design was used: age, gender, ethnicity, and home locality were entered in step 1 as control variables, and the five impulsivity dimensions with significant group differences (i.e., UPPSP Negative Urgency and Positive Urgency, BIS-11 Motor Impulsiveness, Attentional Impulsiveness and Non-planning Impulsiveness) were entered in step 2. Data in Table 3 revealed that UPPSP Negative Urgency positively predicted at-risk gambling (OR = 1.095, *p* < 0.01) in the non-problem gambling *VS* at-risk gambling model, and that Negative Urgency also positively predicted problem gambling (OR = 1.098, *p* < 0.05) in the non-problem gambling *VS* problem gambling model, but Negative Urgency did not have a significant main effect in the at-risk gambling *VS* problem gambling model. Besides that, none of the UPPSP Positive Urgency, BIS-11 Motor Impulsiveness, Attentional Impulsiveness and Non-planning Impulsiveness displayed a significant predictive effect on gambling behaviors in any of the models.

## Discussion

In this study, we contrasted different dimensions of impulsivity in young adults with varying severity of gambling (i.e., no PG, at-risk PG, and PG). Our data revealed elevated scores on UPPSP Negative Urgency and Positive Urgency as well as BIS-11 Motor Impulsiveness and Attentional Impulsiveness in both PGs and ARGs compared with NPGs. PGs also had higher scores than NPGs on BIS-11 Non-planning Impulsiveness, and had higher scores than ARGs on UPPSP Positive Urgency. Significant positive associations were found between SOGS scores and Negative Urgency, Positive Urgency, Motor Impulsiveness, Attentional Impulsiveness, and Non- planning Impulsiveness scores. More interesting, Negative Urgency positively predicted both at-risk PG and PG compared with NPG, while it did not have a similar effect in the at-risk PG *VS* PG model. These results support our hypotheses that different dimensions of impulsivity are distinguishingly involved in gambling disorder, and specific trait (i.e., Negative Urgency) is prior to the development of overt pathology rather than as a result of the pathology itself, putatively representing a vulnerability candidate of gambling disorder.

Despite its multidimensional nature, impulsivity is considered a core characteristic of gambling disorder, and clinical individuals with PG have been characterized by increased self-reported and neurocognitive impulsivity^9^,^25^. With regard to self-reported impulsivity traits, a recent study using exploratory factor analyses found higher BIS-11 Motor Impulsiveness, Attentional Impulsiveness, and Non-planning Impulsiveness in PGs versus healthy controls^27^. Elevated dimensions of impulsivity (i.e., Negative Urgency, Positive Urgency, Motor Impulsiveness, Attentional Impulsiveness, and Non-planning Impulsiveness) in our non-clinical sample of PG are in keeping with the present literature^27^,^31^,^67^,^68^. Furthermore, at-risk PG individuals (i.e., ARGs) in our study also showed higher levels of these traits (except Non-planning Impulsiveness), while ARGs and PGs were not differentiated on these dimensions of impulsivity (except Positive Urgency), which supports the notion that elevated impulsiveness in PG (Negative Urgency, Motor Impulsiveness, Attentional Impulsiveness in the present study) may not stem from the disorder itself as the harmful effects of recurrent gambling^52^. Although in our study PGs did have higher scores on Positive Urgency than ARGs (as well as the fact that the partial correlation between Positive Urgency and SOGS score was significantly positive with a small degree), Positive Urgency did not demonstrate any main effects as a predictor on distinguishing PG from at-risk PG behaviors in the regression model. More powerful evidence should be further gathered to clarify on this issue. In addition, our data of the Delay-discounting Test (DDT) revealed no significant group differences between the PGs, ARGs and NPGs, inconsistent with previous reports showing steeper delay discounting in PGs^31^,^40^,^69^,^70^, which could be due to different methodologies and subject samples, thus cross-cultural studies should be of help to investigate the divergence of results with universal measurements.

More important findings in this study were from the logistic regression models demonstrating that Negative Urgency positively predicted both ARG and PG compared with NPG (i.e., as a common factor in varying forms of gambling), but without a similar trend in the model predicting PG compared with ARG (i.e., as a gambling- induced harmful effect). Up to now, this is the first study with direct evidence in non-treatment seeking populations showing that specific trait of impulsivity (Negative Urgency) is overtly increased in both at-risk PG and PG as a predictive indicator. The data suggest that Negative Urgency as a personality trait of impulsivity probably predates gambling disorder, rather than as a consequence of the pathology. Negative Urgency refers to the tendency to experience strong impulses under condition of negative affect^60^ and has been regarded as an aspect of the inhibitory process^71^. Previous studies found that PGs show significant increases in Negative Urgency^31^,^35^,^68^, and a meta-analysis of UPPSP impulsivity model in PG concluded that Negative Urgency might be a risk factor for the etiology of gambling disorder^54^. Our results of elevated Negative Urgency implicated in both at-risk PG and PG increase knowledge to the current literature. More importantly, these findings support the hypothesis that Negative Urgency is prior to the development of PG, putatively representing a vulnerability marker for gambling disorder. In view of the common neurobiological bases between PG and drug addiction, our findings also call into further research on the underlying roles of Negative Urgency in the development of substance use disorders.

Several limitations should be noted in this study. Firstly, the study design was cross-sectional in nature, so we were not able to determine causal relationships between the impulsivity traits and gambling disorder. Although the data suggest that Negative Urgency may increase risk for PG, future longitudinal research are warranted. Secondly, gambling status and impulsivity traits were assessed using self-reported measurements, which may be subject to bring bias into the analyses. Objective assessments should be incorporated in future studies. Thirdly, the study participants consisted of young adult college students, therefore the findings could not be generalized to the entire population of PGs and other age groups. Future work should examine the differences on impulsivity models between different gambling samples (e.g., college students, community gamblers, clinical patients). Besides, increasing studies have featured a trend toward decreased discounting of probabilistic rewards (i.e., more shallow probability discounting) in PG, suggesting a reduction in risk aversion^72^,^73^. Unfortunately, probability discounting (PD) was not investigated in our study. The relationships between PD and gambling should be further tested.

Despite these limitations, our results indicate that Negative Urgency, Positive Urgency, Motor Impulsiveness, and Attentional Impulsiveness are increased in PGs and ARGs, and moreover, Negative Urgency is a common factor in predicting both at-risk PG and PG but not affected by the severity of gambling, putatively identified as a vulnerability candidate of gambling disorder. The findings may help to further understand the pathway of specific impulsivity traits implicated in the development of gambling disorder, and promote the development of effective prevention and early interventions of problematic gambling behaviors.

## Additional Information

**How to cite this article:** Yan, W.-S. *et al*. Comparison of impulsivity in non-problem, at-risk and problem gamblers. *Sci. Rep.* **6**, 39233; doi: 10.1038/srep39233 (2016).

**Publisher's note:** Springer Nature remains neutral with regard to jurisdictional claims in published maps and institutional affiliations.

## Figures and Tables

**Table 1:** Demographic characteristics and impulsivity scores of the sample (*N* = 1120).

Variables	Non-problem gamblers (NPGs, n = 978)	At-risk gamblers (ARGs, n = 87)	Problem gamblers (PGs, n = 55)
Age, years (*M* ± *SD*)	19.09 ± 1.03	19.29 ± 1.07	19.55 ± 1.02
Gender, Male n (%)	250 (25.6)	37 (42.5)	34 (61.8)
Ethnicity, Hans n (%)	609 (62.3)	55 (63.2)	27 (49.1)
Home locality, Urban n (%)	230 (23.5)	19 (21.8)	8 (14.5)
FTND score (*M* ± *SD*)	0.05 ± 0.33	0.18 ± 0.77	0.36 ± 1.09
AUDIT score (*M* ± *SD*)	1.76 ± 2.07	2.93 ± 2.45	3.27 ± 2.45
SOGS score (*M* ± *SD*)	0.33 ± 0.63	3.36 ± 0.48	6.80 ± 1.98
UPPSP score (*M* ± *SD*)			
Sensation Seeking	27.85 ± 6.31	28.77 ± 7.04	29.45 ± 7.17
Lack of Perseverance	20.93 ± 3.97	21.11 ± 4.01	21.71 ± 3.34
Lack of Premeditation	22.55 ± 4.88	22.11 ± 4.82	23.24 ± 5.49
Negative Urgency	25.97 ± 5.59	28.16 ± 5.49	29.51 ± 6.05
Positive Urgency	27.67 ± 6.50	29.48 ± 7.03	31.69 ± 7.04
BIS-11 score (*M* ± *SD*)			
Motor Impulsiveness	20.25 ± 3.28	21.01 ± 3.31	21.85 ± 3.32
Attentional Impulsiveness	16.83 ± 3.33	17.43 ± 3.59	18.07 ± 3.14
Non-planning Impulsiveness	28.89 ± 4.49	29.37 ± 4.77	29.65 ± 4.88
DDT score (*M* ± *SD*)			
*k*_gain_/log-transformed	0.30 ± 0.25/−0.73 ± 0.49	0.31 ± 0.27/−0.67 ± 0.42	0.32 ± 0.23/−0.68 ± 0.48
*k*_loss_/log-transformed	0.24 ± 0.23/−0.91 ± 0.56	0.25 ± 0.24/−0.87 ± 0.55	0.33 ± 0.23/−0.69 ± 0.53

Note. FTND = Fagerström Test for Nicotine Dependence, AUDIT = Alcohol Use Disorders Identification Test, SOGS = South Oaks Gambling Screen, UPPSP = UPPSP Impulsive Behaviors Scale, BIS = Barratt Impulsiveness Scale, DDT = Delay-discounting Test, and *k* represents the discounting rate.

**Table 2:** Partial correlations (*r*_*p*_) between impulsivity measures and SOGS scores (*N* = 1120).

Variables	1	2	3	4	5	6	7	8	9	10
1. SOGS score	—									
2. UPPSP Sensation Seeking	0.070	—								
3. UPPSP Lack of Perseverance	0.067	0.186***	—							
4. UPPSP Lack of Premeditation	0.055	0.145***	0.630***	—						
5. UPPSP Negative Urgency	0.213***	0.077*	0.321***	0.263***	—					
6. UPPSP Positive Urgency	0.176***	0.196***	0.216***	0.166***	0.745***	—				
7. BIS Motor Impulsiveness	0.155***	0.046	0.324***	0.375***	0.447***	0.409***	—			
8. BIS Attentional Impulsiveness	0.145***	0.036	0.485***	0.353***	0.492***	0.382***	0.522***	—		
9. BIS Non-planning Impulsiveness	0.112***	0.156***	0.585***	0.592***	0.446***	0.317***	0.480***	0.511***	—	
10. DDT *k*_gain_ (log-transformed)	0.018	0.048	0.023	0.052	0.002	0.022	0.030	0.013	0.010	—
11. DDT *k*_loss_ (log-transformed)	0.045	0.039	0.023	0.007	0.013	0.025	0.016	0.021	0.027	0.362***

Note. SOGS = South Oaks Gambling Screen, UPPSP = UPPSP Impulsive Behaviors Scale, BIS = Barratt Impulsiveness Scale-11, DDT = Delay-discounting Test, and *k* represents the discounting rate. Control variables: age, gender, ethnicity, and home locality.

^*^p < 0.05, ^**^p < 0.01, ^***^p < 0.001.

**Table 3:** Logistic regression analyses of impulsivity scores on gambling behavior controlling for demographics.

Models	NPG *VS* ARG^a^ (At-risk Gambling = 1)			NPG *VS* PG^b^ (Problem Gambling = 1)			ARG *VS* PG^c^ (Problem Gambling = 1)		
	B	Wald χ^2^	OR (95% CI)	B	Wald χ^2^	OR (95% CI)	B	Wald χ^2^	OR (95% CI)
**Step 1**									
Age	0.151	2.062	1.163 (0.946–1.429)	0.308	5.749*	1.360 (1.058–1.749)	0.261	2.150	1.299 (0.916–1.842)
Gender (Male = 1)	0.885	13.735**	2.422 (1.517–3.867)	1.678	29.926***	5.355 (2.935–9.769)	0.854	4.921*	2.348 (1.104–4.991)
Ethnicity (Hans = 1)	0.027	0.013	1.028 (0.645–1.638)	−0.464	2.462	0.629 (0.352–1.122)	−0.569	2.242	0.566 (0.269–1.192)
Home locality (Urban = 1)	−0.010	0.001	0.990 (0.567–1.727)	−0.519	1.472	0.595 (0.257–1.377)	−0.372	0.530	0.689 (0.253–1.877)
**Step 2**									
UPPSP Negative Urgency	**0.091**	8.072**	**1.095 (1.029–1.166)**	**0.093**	5.034*	**1.098 (1.012–1.191)**	0.032	0.379	1.032 (0.933–1.143)
UPPSP Positive Urgency	−0.018	0.520	0.983 (0.937–1.031)	0.015	0.232	1.016 (0.954–1.082)	0.032	0.749	1.033 (0.960–1.111)
BIS Motor Impulsiveness	0.033	0.579	1.033 (0.950–1.125)	0.093	2.978	1.097 (0.987–1.219)	0.062	0.908	1.064 (0.936–1.210)
BIS Attentional Impulsiveness	−0.005	0.013	0.995 (0.913–1.084)	0.019	0.114	1.019 (0.912–1.140)	0.064	0.895	1.067 (0.933–1.219)
BIS Non-planning Impulsiveness	−0.012	0.136	0.989 (0.930–1.051)	−0.029	0.536	0.971 (0.898–1.050)	−0.056	1.136	0.946 (0.853–1.048)

Note. NPG = Non-problem Gambling, ARG = At-risk Gambling, PG =  Problem Gambling, CI = confidence interval, OR = odds ratio, UPPSP = UPPSP Impulsive Behaviors Scale, BIS = Barratt Impulsiveness Scale-11.

^a^N = 1065, Nagelkerke R^2^ = 0.063.

^b^N = 1033, Nagelkerke R^2^ = 0.196.

^c^N = 142, Nagelkerke R^2^ = 0.152.

^*^p < 0.05. ^**^p < 0.01, ^***^p < 0.001.
